# Evidence-based medication adherence among seniors in the first year after heart failure hospitalisation and subsequent long-term outcomes: a restricted cubic spline analysis of adherence-outcome relationships

**DOI:** 10.1007/s00228-023-03467-7

**Published:** 2023-02-28

**Authors:** Xiwen Qin, Joseph Hung, Matthew W. Knuiman, Tom G. Briffa, Tiew-Hwa Katherine Teng, Frank M. Sanfilippo

**Affiliations:** 1 School of Population and Global Health, University of Western Australia, Perth, WA Australia; 2 Medical School, University of Western Australia, Perth, WA Australia; 3 National Heart Centre Singapore, Singapore, Singapore

**Keywords:** Heart failure, Medication adherence, Renin-angiotensin system inhibitors, β-blockers, Outcomes, Restricted cubic splines

## Abstract

**Purpose:**

Non-adherence to heart failure (HF) medications is associated with poor outcomes. We used restricted cubic splines (RCS) to assess the continuous relationship between adherence to renin-angiotensin system inhibitors (RASI) and β-blockers and long-term outcomes in senior HF patients.

**Methods:**

We identified a population-based cohort of 4234 patients, aged 65–84 years, 56% male, who were hospitalised for HF in Western Australia between 2003 and 2008 and survived to 1-year post-discharge (landmark date). Adherence was calculated using the proportion of days covered (PDC) in the first year post-discharge. RCS Cox proportional-hazards models were applied to determine the relationship between adherence and all-cause death and death/HF readmission at 1 and 3 years after the landmark date.

**Results:**

RCS analysis showed a curvilinear adherence-outcome relationship for both RASI and β-blockers which was linear above PDC 60%. For each 10% increase in RASI and β-blocker adherence above this level, the adjusted hazard ratio for 1-year all-cause death fell by an average of 6.6% and 4.8% respectively (trend *p* < 0.05) and risk of all-cause death/HF readmission fell by 5.4% and 5.8% respectively (trend *p* < 0.005). Linear reductions in adjusted risk for these outcomes at PDC ≥ 60% were also seen at 3 years after landmark date (all trend *p* < 0.05).

**Conclusion:**

RCS analysis showed that for RASI and β-blockers, there was no upper adherence level (threshold) above 60% where risk reduction did not continue to occur. Therefore, interventions should maximise adherence to these disease-modifying HF pharmacotherapies to improve long-term outcomes after hospitalised HF.

## Introduction

Rates of mortality and hospitalisations for patients with heart failure (HF) remain high despite significant advances in HF pharmacotherapies [1, 2]. Renin-angiotensin system inhibitors (RASI) and β-blockers have proven prognostic benefit and are recommended by guidelines in all patients with HF and reduced ejection fraction (HFrEF), unless contraindicated or not tolerated [2, 3]. Non-adherence to these disease-modifying pharmacotherapies in HF patients is associated with increased mortality and hospitalisation risk as well as healthcare costs [4–7]. Hospitalised HF patients may be especially prone to non-adherence because they are often elderly and have multiple comorbid conditions [8, 9]. Hence, medication non-adherence remains a significant obstacle to enhancing effectiveness of guideline-based pharmacotherapies in HF [10].

Estimates of adherence to RASIs and β-blockers in HF patients vary considerably, ranging from 40 to over 90% depending on the method of estimating adherence, length of observation and the cohort characteristics [5, 6, 11–14]. Studies of adherence using pharmacy claims data have mostly calculated the proportion of days covered (PDC) for each medication [15, 16] and categorised a PDC threshold of 80% as being sufficiently adherent without testing if this cut-off was associated with optimal outcomes [4, 7, 11, 13, 16, 17].

The validity of using arbitrary PDC cut-offs as against evidence-based thresholds has been challenged [18, 19]. It is also likely that optimal adherence levels will vary according to diseases, medications and patient characteristics [18–20]. In fact, a single longitudinal study suggested that a medication adherence above 88% was necessary for optimal event-free survival in HF patients [21]. An alternative strategy for exploring the association without assumption of linearity is to use restricted cubic splines (RCS) [22]. We have previously reported that PDC calculated from administrative drug databases was the most consistent predictor of subsequent mortality in a HF cohort, and that RCS could be used to assess the adherence-outcome relationship across the continuous adherence scale without assumption of linearity or adherence thresholds [23]. The aim of this study was to apply RCS analysis in a population-based cohort of seniors, aged 65–84 years, to evaluate the continuous relationship between PDC adherence to RASI and β-blockers in the first year after HF hospitalisation and subsequent all-cause death and/or HF readmission over a 3-year follow-up period.

## Material and methods

### Data sources

This study used government-held administrative databases, regularly audited for quality, to create person-linked health records as previously described [24, 25]. Briefly, the Hospital Morbidity Data Collection from the Western Australian Data Linkage System was used to identify patients with hospitalisation for HF from 1 January 2003 to 31 December 2008, and linked to matching death records from the Western Australian death registry [24, 25]. Pharmaceutical Benefits Scheme (PBS) claims data were used to identify matching records for the dispensing of RASI or β-blockers approved for HF (bisoprolol, carvedilol, metoprolol tartrate, metoprolol succinate and nebivolol) using their Anatomical Therapeutic Chemical (ATC) codes between June 2002 and June 2011 in concessional health card holders [12, 23, 26].

### Study cohort

**Fig. 1:**
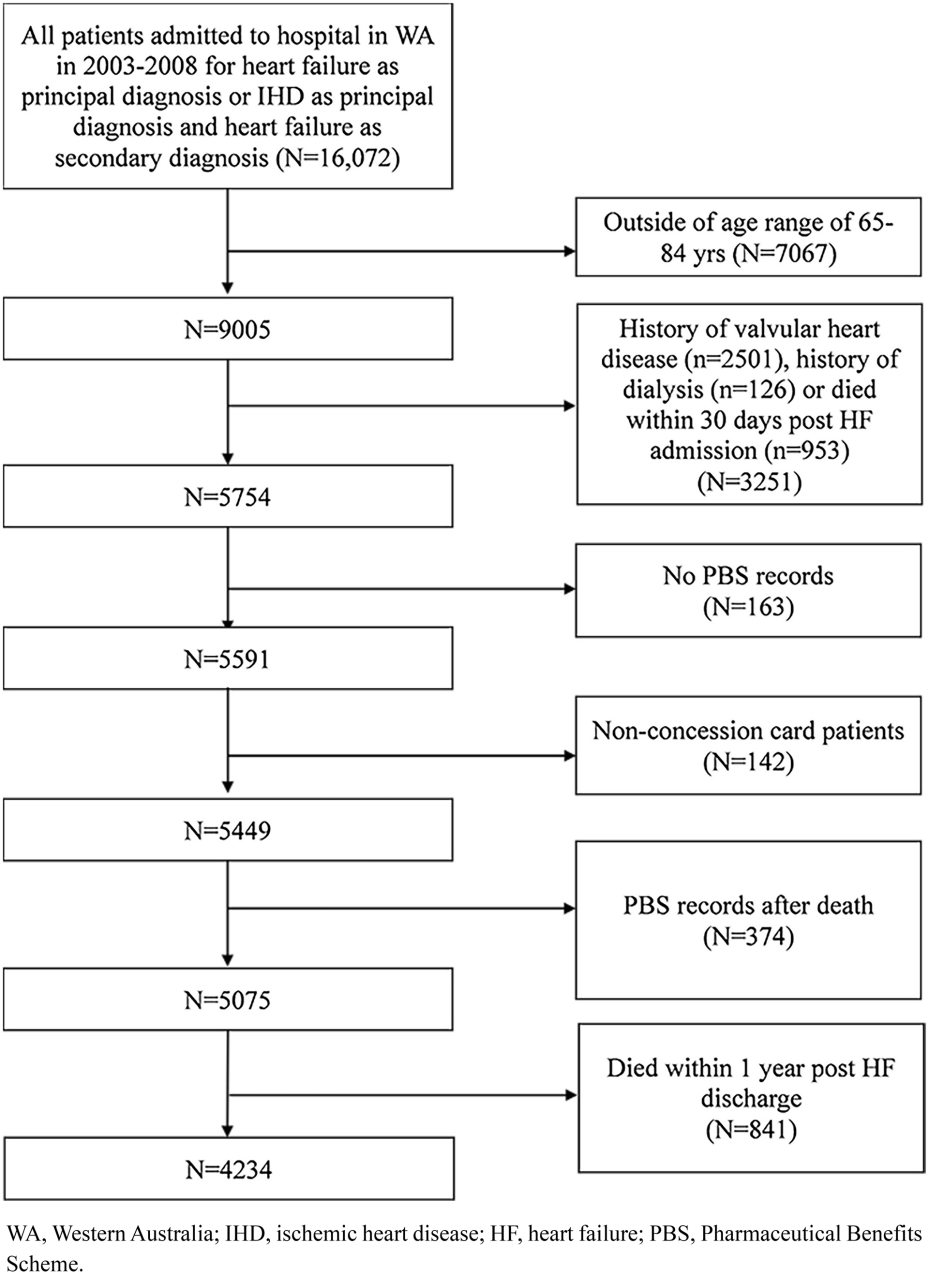
Flowchart of patient selection for the study cohort. WA, Western Australia; IHD, ischemic heart disease; HF, heart failure; PBS, Pharmaceutical Benefits Scheme

Figure 1 shows the patient selection flowchart for the study cohort. We identified a cohort of 4234 seniors, aged 65–84 years, with an index (first-in-period) hospitalisation for HF in 2003–2008 as a principal diagnosis (International Classification of Diseases and Related Health Problems 10th revision Australian Modification code I50) or HF as a secondary diagnosis and ischemic heart disease (IHD) as a principal diagnosis. The method used to identify the cohort and to identify HF and other comorbidities has been previously described [12, 23]. The coded hospital discharge diagnosis of HF has been previously validated by medical chart review [27]. Patients with a history of valvular heart disease or renal dialysis, non-concession card holders and those without any PBS records were excluded [12, 23]. All patients had to survive to 1 year following the date of hospital discharge (designated landmark date) in order to measure their medication adherence over a 12-month period.

### Data collection

The study methodology has been described previously [23, 25]. Demographic data were identified based on the index HF admission, and residential location was used to derive the Accessibility Remoteness Index of Australia (ARIA +) classification which measures relative access to services [28]. The Socio-Economic Indexes for Areas (SEIFA) [29], included as the index of relative socio-economic disadvantage, was assigned to each patient based on their residential postcode and grouped into quintiles, with the first quintile representing the most disadvantaged group and last quintile representing the least disadvantaged. Comorbid conditions were identified from the Hospital Morbidity Data Collection dataset using a fixed 20-year look-back period from the landmark date. Prevalent HF was defined as any HF hospitalisation prior to the index admission. The Charlson comorbidity index (CCI) was derived from the identified comorbidities with exclusion of HF [30]. Individual medications including RASI and β-blockers were identified from the PBS data by their Anatomical Therapeutic Chemical code [23, 26].

### Medication adherence

We estimated adherence to RASIs and HF-approved β-blockers in the 1-year landmark period after their index hospital discharge. Users of RASI and β-blockers were required to have 2 or more supplies of the drug class in order to calculate the adherence measure (PDC) more precisely [12, 23]. Medication adherence estimates were calculated for RASI and β-blockers using the PDC method as previously described [15, 23]. The following equation was used to calculate the PDC for each patient for each drug group:

$$\mathrm{PDC}=\frac{\sum\mathrm{days}\;\mathrm{covered}\;\mathrm{by}\;\mathrm{the}\;\mathrm{medications}\;\geq\;2\;\mathrm{supplies}\;\mathrm{available}}{\sum\mathrm{days}\;\mathrm{from}\;\mathrm{first}\;\mathrm{supply}\;\mathrm{to}\;\mathrm{one}\;\mathrm{year}\;\mathrm{landmark}\;\mathrm{date}}\times100\%$$

Thus, the PDC is the proportion of days that are covered by the drug supply during the landmark period from first supply to the 1-year landmark date. The PDC only counts once the days with overlapping supplies, so that its value never exceeds 100%. If patients were hospitalised during the landmark period, we assumed they had full adherence during the period of hospitalisation.

Dosing information is not captured in PBS data, so we checked the registered product information for each drug and assumed that RASI were dosed at one per day, except for enalapril (two per day) and captopril (three per day), and β-blockers were dosed at one per day except for metoprolol and carvedilol which were assumed to be two per day. To allow for possible gaps in drug use, the expected duration of supply was estimated separately for each RASI and β-blocker from the 75th percentile of the distribution of time to next supply date [31]. The 75th percentile was 35 days for all RASI drugs, 35 days for metoprolol succinate, nebivolol and carvedilol, and 50 days for metoprolol tartrate, which was consistent with PBS prescriptions which are intended to be approximately 1-month supplies with exception of metoprolol tartrate.

We examined PDC as a continuous as well as categorical variable using PDC ≥ 90% to denote a near full adherence. Use of other cardio-active medications including mineralocorticoid receptor antagonists (MRA), diuretics, anti-arrhythmic agents, statins and calcium channel blockers was captured as separate binary variables for each drug group, defined as at least two scripts filled within the landmark period.

### Study outcomes

We used landmark analysis to correct for the immortal time bias inherent in the analysis of time-to-event outcomes between groups where adherence is determined during the year following index admission [32]. The primary outcome was time to all-cause death at 1 and 3 years after the landmark date. Secondary outcomes were time to first non-fatal HF readmission (principal diagnosis) and composite of all-cause death/HF readmission, whichever occurred first.

### Statistical methods 

Descriptive statistics were presented as mean with standard deviation (SD) for normally distributed continuous variables and frequency (%) for categorical variables. We tested differences between groups using the *t*-test for continuous variables and chi-squared test for categorical variables. Time to all-cause death, first HF readmission and death/HF readmission were plotted using a cumulative incidence function and Gray’s test used to assess for differences between adherence groups [33].

For each drug group, we fitted Cox proportional hazards models with RCS to investigate the effect of adherence to that drug group on outcomes [20, 22, 23]. All Cox models were adjusted for baseline demographics, comorbidities and concomitant medication use (see variables in Table 1). This included adjustment in RAS users for concomitant β-blocker adherence (PDC ≥ 90% or < 90%) and conversely concomitant RASI adherence in β-blocker users. RCS Cox models revealed the shape of the relationship between continuous PDC adherence and outcomes without a priori assumptions of linearity [22]. The RCS method fits a smooth continuous curve of adjusted HRs with 95% confidence intervals (CI) across adherence levels, allowing for cubic form changes at arbitrary knot points (30%, 60%, 80%), and a linear form at the tail ends. The RCS plots were restricted to PDC ≥ 30% due to small frequencies below this level. A PDC of 90% was chosen as the reference value for calculation of adjusted HRs to compare against a near full adherence level. The RCS plots were used to visually and statistically assess the continuous adherence-outcome relationship.

**Table 1:** Characteristics and crude outcomes of patients surviving 1-year post-HF hospitalisation, and in subgroups of RASI and β-blocker users stratified by adherence level (PDC ≥ 90% versus PDC < 90%)

**Characteristic**	**Total (*n* = 4234)**	**RASI (*n* = 3668)**		***p*-value**	**β-blockers (*n* = 2822)**		***p*-value**
		**PDC < 90%**	**PDC ≥ 90%**		**PDC < 90%**	**PDC ≥ 90%**	
Number	4234	1580 (43.1)	2088 (56.9)	NA	1745 (61.8)	1077 (38.2)	NA
Sex							
Male	2365 (55.9)	911 (57.7)	1164 (55.8)	0.38	983 (56.3)	651 (60.5)	0.03
Age (mean ± SD)	76.4 (5.5)	76.3 (5.5)	76.3 (5.4)	0.95	76.5 (5.5)	75.4 (5.4)	< 0.0001
Age group							
65–69	677 (16.0)	259 (16.4)	333 (15.6)	0.96	278 (15.9)	211 (19.6)	< 0.0001
70–74	929 (21.9)	357 (22.6)	469 (22.5)		385 (22.1)	284 (26.4)	
75–79	1257 (29.7)	466 (29.5)	631 (30.2)		507 (29.1)	309 (28.7)	
80–84	1371 (32.4)	498 (31.5)	655 (31.4)		575 (33.0)	273 (25.4)	
Indigenous status	95 (2.2)	45 (2.9)	27 (1.3)	0.001	33 (1.9)	13 (1.2)	0.16
Private insurance	1153 (27.2)	383 (24.2)	616 (29.5)	0.001	448 (25.7)	333 (30.9)	0.003
ARIA + classification				0.01			0.04
Major city	1942 (45.9)	796 (50.4)	1015 (48.6)		899 (51.5)	520 (48.3)	
Inner regional	1250 (29.5)	457 (28.9)	681 (32.6)		525 (30.1)	361 (33.5)	
Outer regional	445 (10.5)	185 (11.7)	239 (11.5)		197 (11.3)	117 (10.9)	
Remote	195 (4.6)	79 (5.0)	103 (4.9)		68 (3.9)	51 (4.7)	
Very remote	140 (3.3)	63 (4.0)	50 (2.4)		56 (3.2)	28 (2.6)	
SEIFA				0.02			0.77
First quintile (most disadvantage)	319 (7.5)	134 (8.5)	134 (6.4)		123 (7.1)	77 (7.2)	
Second quintile	858 (20.3)	319 (20.3)	420 (20.2)		356 (20.4)	213 (19.9)	
Third quintile	731 (17.3)	290 (18.3)	346 (16.6)		310 (17.8)	175 (16.3)	
Fourth quintile	1004 (23.7)	348 (22.0)	526 (25.2)		416 (23.8)	258 (24.0)	
Fifth quintile (least disadvantage)	1322 (31.2)	489 (30.9)	662 (31.6)		540 (31.0)	354 (32.9)	
HF hospitalisation prior to index admission	1325 (31.3)	537 (34.0)	620 (30.0)	0.006	547 (31.4)	338 (31.4)	0.98
HF readmission within landmark period	805 (19.0)	389 (24.6)	336 (16.1)	< 0.001	402 (23.0)	183 (17.0)	0.0001
Comorbidities							
IHD	3102 (73.3)	1195 (75.6)	1564 (74.9)	0.62	1430 (82.0)	854 (79.3)	0.08
Hypertension	3288 (77.7)	1293 (81.8)	1631 (78.1)	0.001	1433 (82.1)	860 (80.0)	0.14
AF	1963 (46.4)	758 (48.0)	957 (45.8)	0.19	854 (48.9)	513 (47.6)	0.50
Diabetes	1765 (41.7)	703 (44.5)	877 (42.0)	0.14	753 (43.2)	479 (44.5)	0.49
COPD	1319 (31.2)	543 (34.4)	576 (27.6)	< 0.001	470 (26.9)	226 (21.0)	< 0.0001
CKD	1496 (35.3)	637 (40.3)	673 (32.2)	< 0.001	733 (42.0)	363 (33.7)	< 0.0001
PVD	760 (18.0)	305 (19.3)	357 (17.1)	0.08	351 (20.1)	187 (17.4)	0.07
Stroke	522 (12.3)	209 (13.2)	240 (11.5)	0.12	245 (14.0)	117 (10.9)	0.01
Depression	339 (8.0)	123 (7.8)	142 (6.8)	0.26	130 (7.5)	60 (5.6)	0.05
Dementia	190 (4.5)	62 (3.9)	91 (4.4)	0.51	72 (4.1)	22 (2.0)	0.002
CCI score categories							
0	941 (22.2)	280 (17.7)	523 (25.1)	< 0.001	366 (21.0)	272 (25.3)	0.002
1–2	1423 (33.6)	542 (34.3)	693 (33.2)		571 (32.7)	350 (32.5)	
3–4	919 (21.7)	357 (22.6)	441 (21.1)		370 (21.2)	244 (22.7)	
> 4	951 (22.5)	401 (25.4)	431 (20.6)		438 (25.1)	211 (19.5)	
Other drugs in landmark period							
RASI	3668 (86.6)	NA	NA	NA	1575 (90.3)	997 (92.6)	0.03
β-blockers	2822 (66.7)	1078 (68.2)	1494 (71.6)	0.03	NA	NA	NA
MRA	1350 (31.9)	570 (36.1)	666 (31.9)	0.06	564 (32.3)	412 (38.3)	0.001
Digoxin	1102 (26.0)	421 (26.7)	554 (26.5)	0.94	442 (25.3)	316 (29.3)	0.02
Loop diuretics	3595 (84.9)	1402 (88.7)	1800 (86.2)	0.03	1494 (85.6)	930 (86.4)	0.58
Warfarin	1245 (29.4)	479 (30.3)	635 (30.4)	0.95	534 (30.6)	376 (34.9)	0.02
Anti-arrhythmic	532 (12.6)	232 (14.7)	257 (12.3)	0.04	249 (14.3)	130 (12.1)	0.10
Statins	2813 (66.4)	1059 (67.0)	1475 (70.8)	0.01	1303 (74.7)	818 (76.0)	0.44
CCB	911 (21.5)	321 (20.3)	463 (22.2)	0.17	395 (22.6)	177 (16.4)	< 0.001
Crude 1-year outcome post landmark date							
All-cause death	581 (13.7)	258 (16.3)	241 (11.5)	< 0.001	253 (14.5)	101 (9.4)	0.001
HF readmission	518 (12.2)	238 (15.1)	234 (11.2)	< 0.001	253 (14.5)	109 (10.1)	0.001
All-cause death/HF readmission	968 (22.9)	422 (26.7)	432 (20.7)	< 0.001	442 (25.3)	188 (17.5)	0.001
Crude 3-year outcome post landmark date							
All-cause death	1466 (34.6)	612 (38.7)	637 (30.5)	< 0.001	609 (34.9)	295 (27.4)	0.002
HF readmission	1028 (24.3)	439 (27.8)	487 (23.3)	< 0.001	455 (26.1)	255 (23.7)	0.11
All-cause death/HF readmission	2011 (47.5)	831 (52.6)	911 (43.6)	< 0.001	847 (48.5)	448 (41.6)	0.003

*SD* standard deviation, *PDC* proportion of days covered, *HF* heart failure, *ARIA* +  Accessibility and Remoteness Index of Australia Plus classification, *SEIFA* Socio-Economic Indexes for Areas, *IHD* ischemic heart disease, *AF* atrial fibrillation, *COPD* chronic obstructive pulmonary disease, *CKD* chronic kidney disease, *PVD* peripheral vascular disease *CCI* Charlson comorbidity index, *RASI* renin-angiotensin system inhibitor, *MRA* mineralocorticoid receptor antagonist, *CCB* calcium channel blocker, *NA* not applicable

To compare with results from the Cox proportional hazards models, we also carried out a propensity score (PS) analysis using the inverse probability treatment weighting (IPTW) method to adjust for potential bias in the allocation of patients to adherence groups [34]. The propensity score was estimated using a logistic regression model which included all of the above-mentioned covariates (Table 1) as potential predictors for high adherence (PDC ≥ 90%) to RASI treatment, and likewise a separate model to predict high adherence to β-blockers. A weight was then calculated for each patient as 1/PS in the high adherence group and 1/1-PS for those in the < 90% adherence group. Extreme weight values were truncated at the 5th and 95th percentile ends of the distribution. We confirmed that the IPTW method (through weighting) had adequately balanced the covariate profile of the two groups by comparison of the unweighted and weighted standardised difference in means/proportions for each covariate [34]. We then used weighted Cox regression models that included only the adherence group variable for comparing RASI adherence groups (PDC ≥ 90% vs < 90%), and a separate model for β-blocker adherence groups.

The Akaike information criterion was used to assess the model fit, and the proportional hazards assumptions for the Cox models were tested and showed no violation (*p* > 0.05). For non-fatal HF readmission analysis, we considered death as a competing risk and fitted Cox proportional hazards models to calculate the subdistribution hazard ratios (sHR) [33]. If the RCS showed a linear relationship across the range of PDC values or above a turning point, then in further Cox regression models (without RCS), a continuous linear PDC adherence model was fitted for patients with PDC values in that range. Trend *p*-values were calculated in adjusted Cox regression models to assess the change in risk for each 10% increment in adherence. We also tested for interaction effects between adherence level and sex, age, and concomitant IHD, chronic obstructive pulmonary disease (COPD) or chronic kidney disease (CKD). However, all interaction terms were non-significant (*p* > 0.05), and therefore stratified analysis based on age, sex and disease groups was not required. All statistical analyses were performed with SAS version 9.4 (SAS Institute, Inc. Cary, NC).

## Results

### Baseline characteristics and outcomes 

The characteristics and crude outcomes of the whole study cohort and for subgroups of RASI or β-blocker users stratified by dichotomous adherence levels are shown in Table 1. The study cohort comprised 4234 patients, mean age 76.4 years and 55.9% male, with a mean follow-up time of 30 months (SD 11.3) from landmark date. Among the cohort, 86.6% and 66.7% were using a RASI or β-blocker respectively during the landmark period. Among RASI users, those showing PDC adherence ≥ 90% vs < 90% were less likely to be Indigenous, have prior HF hospitalisation(s) or a HF readmission during landmark period and have hypertension, COPD or CKD as comorbid conditions (Table 1). Among β-blocker users, those showing high adherence (PDC ≥ 90%) were generally younger, more likely male, less likely to experience HF readmission during landmark period and less likely to have COPD, CKD, stroke or dementia as comorbid conditions (Table 1).

### PDC adherence levels

The adherence distribution for RASI and β-blocker users was negatively skewed with a median PDC of 92% (IQR 79–97%) and 82% (IQR 55–95%) respectively. Among RASI and β-blocker users, 56.9% and 38.2%, respectively, achieved a PDC ≥ 90%. At 2 years post-discharge, 83.2% and 84.4% of surviving RASI and β-blocker users, respectively, remained within the same PDC categories (PDC ≥ 90% or < 90%) as in their first year. Among RASI users, 70.1% were also taking a β-blocker and conversely 91.1% of β-blocker users were taking a RASI. There was not a high collinearity between RASI and β-blocker adherence as indicated by a variance inflation factor < 10.

### Cumulative incidence of outcomes according to PDC category

**Fig. 2:**
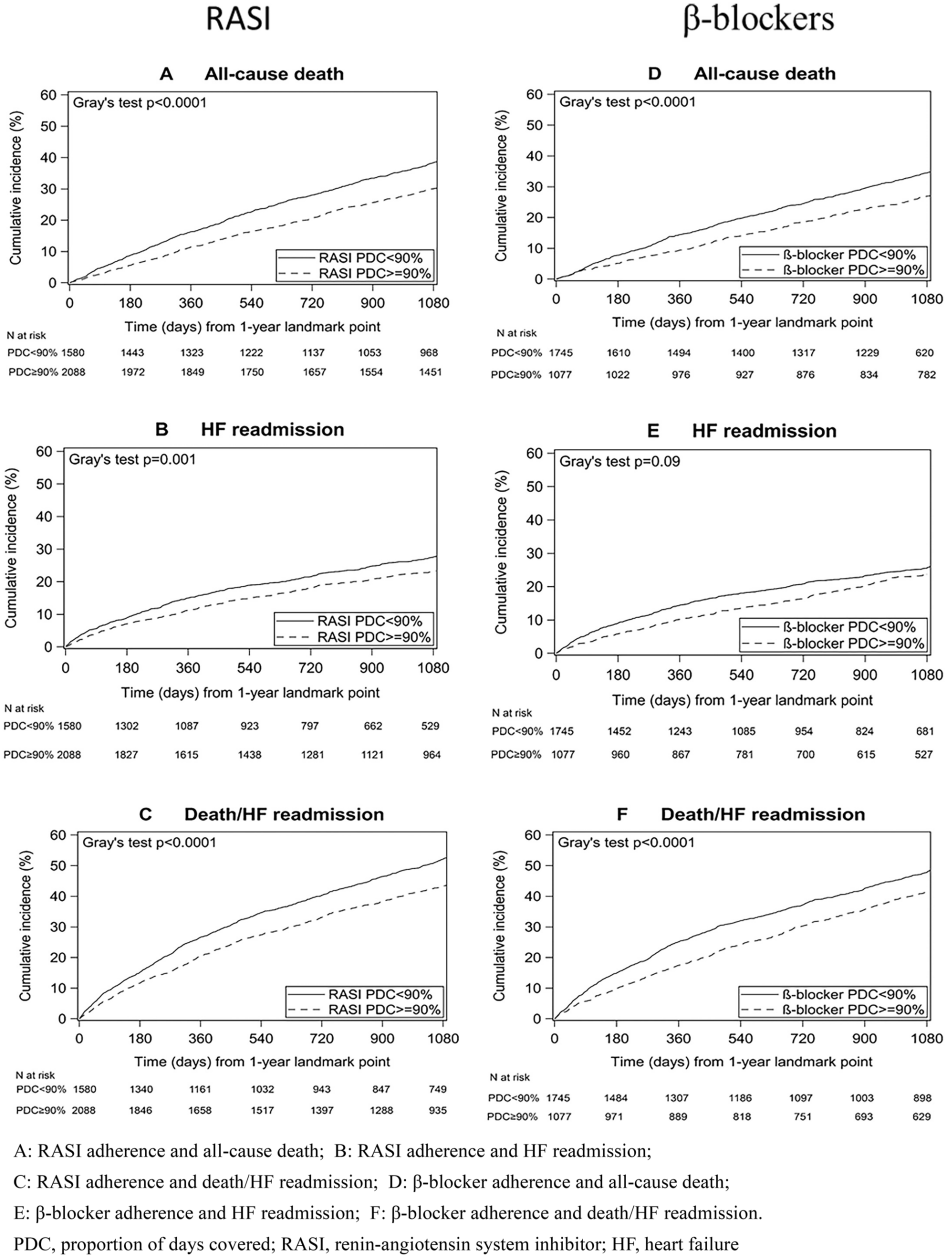
Cumulative incidence function curves for RASI and β-blocker adherence and specified outcomes stratified by proportion of days covered ≥ 90% and < 90%

In the total cohort, crude all-cause death, first non-fatal HF readmission and composite death/HF readmission by 3 years after the landmark date occurred in 34.6%, 24.3% and 47.5% of patients respectively (Table 1). Figure 2A to F show cumulative incidence function curves which demonstrate that in RASI and β-blocker users, a PDC ≥ 90% vs < 90% was significantly associated with a lower incidence of all-cause death, non-fatal HF readmission and death/HF readmission over 3-year follow-up (all *p* ≤ 0.001) but not non-fatal HF readmission in β-blocker users (*p* = 0.09).

### Adjusted hazard ratios according to PDC levels

**Fig. 3:**
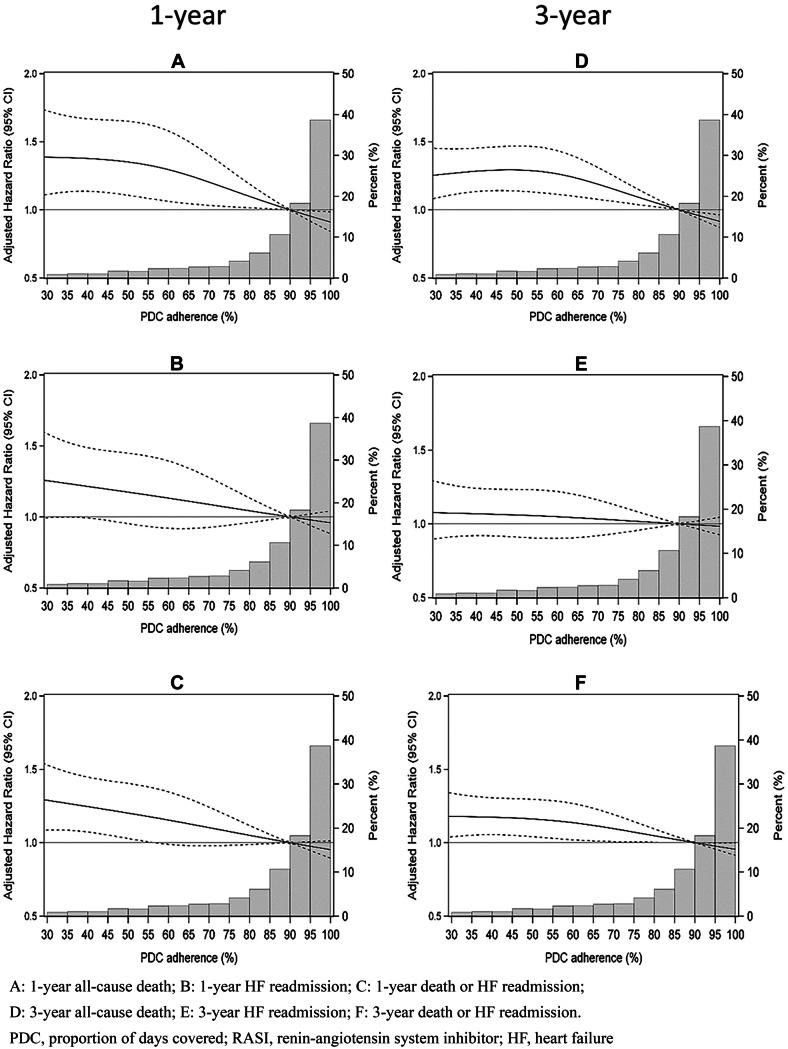
Restricted cubic spline plots from Cox regression models showing adjusted hazard ratios and 95% confidence intervals in RASI users for specified outcomes associated with PDC adherence level. The bars are the frequency distribution of adherence by 5% levels

**Fig. 4:**
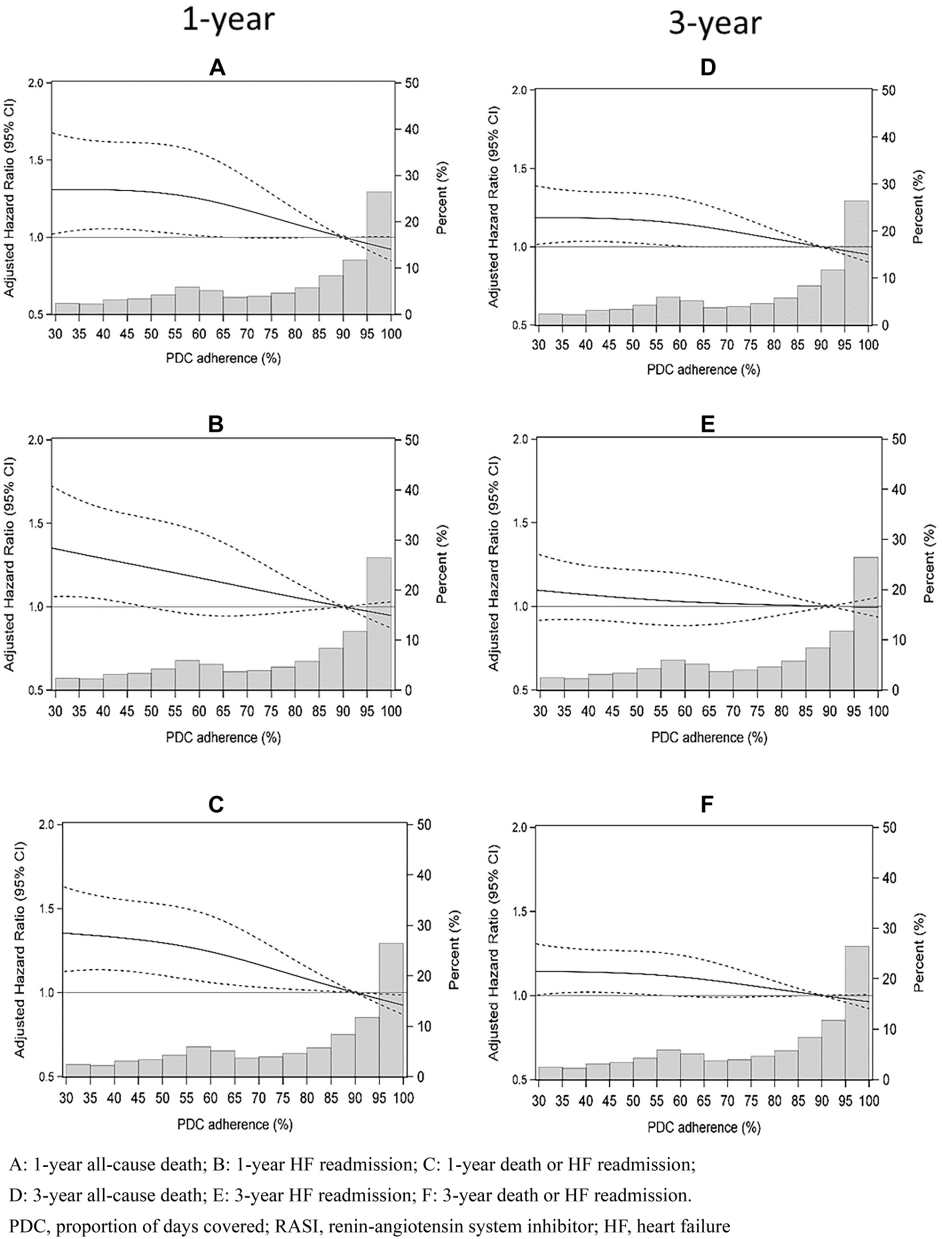
Restricted cubic spline plots from Cox regression models showing adjusted hazard ratios and 95% confidence intervals in β-blocker users for specified outcomes associated with PDC adherence level. The bars are the frequency distribution of adherence by 5% levels

Figures 3 and 4 show Cox regression RCS plots for RASI and β-blocker users respectively with adjusted HRs (or sHRs) and 95% CIs for specified outcomes according to PDC levels. The covariates included in the Cox models for mortality (primary outcome) are indicated in Table 2. The RCS plots for RASI users demonstrated a curvilinear relationship between adherence and all-cause death at 1 and 3 years, with linear reductions in risk at PDC ≥ 60% (Fig. 3A, D). The RCS plots also demonstrated a significant linear relationship between RASI adherence and the composite outcome at 1 and 3 years (Fig. 3C, F). Despite a linear trend, the relationship between RASI adherence and HF readmission at 1 and 3 years was not significant as the 95% CIs crossed unity across the PDC range (Fig. 3B, E).

**Table 2:** Covariate-adjusted and propensity-adjusted hazard ratios and 95% confidence intervals from Cox regression models for 1- and 3-year outcomes after landmark date according to PDC levels for RASI and β-blockers during first year post-HF discharge

	**1-year outcomes**			**3-year outcomes**		
	**All-cause death**	**HF readmission**^**a**^	**Death/HF readmission**	**All-cause death**	**HF readmission**^**a**^	**Death/HF readmission**
**PDC as a categorical variable comparing PDC** ≥ **90% vs < 90% (reference group)**						
**Covariate-adjusted Cox models**^**b**^						
RASI PDC ≥ 90%	0.813 (0.679, 0.972)	0.875 (0.726, 1.054)	0.908 (0.790, 1.010)	0.858 (0.766, 0.961)	0.918 (0.804, 1.047)	0.907 (0.823, 0.998)
* p*-value	0.025	0.159	0.090	0.008	0.203	0.044
**Propensity-adjusted Cox models**^**c**^						
RASI PDC ≥ 90%	0.786 (0.660, 0.937)	0.830 (0.693, 0.993)	0.852 (0.746, 0.974)	0.842 (0.754, 0.940)	0.887 (0.780, 1.008)	0.871 (0.793, 0.956)
* p*-value	0.007	0.042	0.019	0.002	0.067	0.004
**Covariate-adjusted Cox models**^**b**^						
β-blocker PDC ≥ 90%	0.757 (0.597, 0.961)	0.745 (0.591, 0.938)	0.740 (0.621, 0.882)	0.876 (0.759, 1.005)	0.883 (0.754, 1.034)	0.869 (0.773, 0.978)
* p*-value	0.022	0.013	0.0008	0.071	0.123	0.020
**Propensity-adjusted Cox models**^**c**^						
β-blocker PDC ≥ 90%	0.723 (0.583, 0.898)	0.734 (0.592, 0.910)	0.732 (0.622, 0.860)	0.852 (0.747, 0.973)	0.898 (0.774, 1.043)	0.880 (0.788, 0.982)
* p*-value	0.003	0.005	0.0002	0.018	0.158	0.023
**PDC as a continuous variable and with each 10% increase in PDC adherence between 60 and 100%**						
**Covariate-adjusted Cox models**^**b**^						
RASI	0.934 (0.825, 0.976)	0.955 (0.919, 0.994)	0.946 (0.925, 0.980)	0.944 (0.920, 0.970)	0.988 (0.956, 1.014)	0.964 (0.944, 0.988)
Trend *p*-value	0.003	0.031	0.002	< 0.001	0.279	0.001
β-blockers	0.952 (0.922, 0.999)	0.944 (0.911, 0.983)	0.942 (0.920, 0.972)	0.970 (0.946, 0.996)	0.985 (0.952, 1.003)	0.974 (0.950, 0.992)
Trend *p*-value	0.042	0.005	0.001	0.024	0.203	0.019

*PDC *proportion of days covered, *HF *heart failure, *RASI *renin-angiotensin system inhibitor

^a^The hazard ratio for HF readmission is the subdistribution HR treating death as a competing risk event

^b^Cox regression models adjusted for sex, age, indigenous status, private insurance, Accessibility Remoteness Index of Australia plus classification, Socio-Economic Indexes for Areas (as the index of Relative Socio-economic Disadvantage), HF hospitalisation(s) prior to index admission and in landmark period, other comorbidities, Charlson comorbidity index, concurrent medications (yes/no), β-blocker adherence (≥ 90%, < 90%) in RASI model and RASI adherence (≥ 90%, < 90%) in β-blocker model

^c^Inverse probability treatment weighted Cox regression models

The RCS plots for β-blocker users demonstrated a curvilinear relationship between adherence and all-cause death (Fig. 4A, D), and approximately linear relationship with death/HF readmission, particularly at 1 year, with the slope appearing steeper above a PDC of 60% (Fig. 4C, F). Despite a linear trend, the association between β-blocker adherence and HF readmission was not significant, with 95% CIs crossing unity (Fig. 4B, E).

Table 2 shows the covariate-adjusted and the propensity-adjusted (IPTW) HRs for outcomes comparing categorical PDC levels (PDC ≥ 90% vs < 90%). For RASI users, high adherence (PDC ≥ 90%) was associated with a reduced covariate-adjusted HR for 1- and 3-year all-cause death, and 3-year composite death/HF readmission (all *p* < 0.05). For β-blocker users, the covariate-adjusted Cox models showed that a PDC ≥ 90% was associated with a reduced HR for 1-year all-cause death, HF readmission and composite death/HF/readmission (all *p* < 0.05), and also a reduced HR for 3-year death/HF readmission (*p* = 0.020). In general, the IPTW analysis did not materially change the results although the estimated HRs and 95% CIs for both RASI and β-blocker adherence ≥ 90% compared to < 90% were generally lower than those estimated by the covariate-adjusted models (Table 2).

Adherence was further modelled as a continuous variable for PDC between 60 and 100% because the majority of RASI and β-blocker users had PDC values in this range and RCS curves also indicated a more linear risk reduction above 60% PDC (Figs. 3 and 4). Each 10% increase in RASI and β-blocker adherence above this point lowered the adjusted risk for 1-year all-cause death by 6.6% and 4.8% respectively (both trend *p* < 0.05), and the adjusted risk of 1-year all-cause death/HF readmission by 5.4% and 5.8% respectively (both trend *p* < 0.005) (Table 2). A significant linear reduction in risk with increasing RASI and β-blocker adherence levels above 60% PDC was also seen for 3-year all-cause death and composite death/HF readmission (all trend *p* < 0.025) (Table 2).

## Discussion

We evaluated the impact of 1-year adherence to guideline-directed HF medications on long-term mortality and morbidity in a ‘real-world’ population-based cohort of seniors aged ≥ 65 years surviving HF hospitalisation. We observed that adherence to RASI and β-blockers in the year after discharge was suboptimal with only 57% and 38% of users, respectively, achieving near full adherence (PDC ≥ 90%). RCS analysis demonstrated that the pattern of adherence-outcome relationships for both RASI and β-blockers was generally linear above a PDC of 60%. Importantly, the RCS analysis indicated that an empirical adherence threshold of 80% does not provide optimal long-term outcomes because risk continues to reduce above this threshold. Hence, RCS analysis can be used to assess medication adherence as a continuous measure linked to clinical outcomes instead of its conventional use as a binary variable with an arbitrary upper threshold [18, 19].

The observed levels of RASI and β-blocker adherence in our HF cohort are within the range reported for these drug groups in other cohort studies that used a comparable measure of adherence [5, 11, 13, 14]. We also observed similar patient and condition-related factors that have been reported to be associated with medication adherence [9, 17]. The observed lower adherence to β-blockers than RASI may reflect a higher side-effect profile of β-blockers especially in older HF patients. Patients with HF hospitalisation(s) prior to the index admission or during the landmark period were also less likely to be highly adherent to either drug class, but whether this is a cause or effect is unclear. In Australia, medication costs are usually not a barrier to adherence because the majority (≈95%) of seniors are eligible for health concession cards which provide access to PBS-listed drugs at a highly subsidised cost.

Prescription of evidence-based pharmacotherapies is promoted by clinical guidelines, but health outcomes will not improve for patients unless they are adherent to therapy. Poor adherence to proven HF pharmacotherapies, traditionally defined as PDC < 80%, has been associated with an increased risk of all-cause death, hospitalisations and healthcare costs [4–7]. In the present study, we confirmed that high adherence (PDC ≥ 90%) for RASI and β-blockers predicted patients at significantly lower risk of death and a composite of death/HF readmission over 3 years. A systematic review of multi-dimensional interventions to improve medication adherence in HF patients suggests that they can have a significant effect on reducing readmissions and decreasing mortality [6]. However, the level of adherence required to achieve optimal outcomes is unclear as dose–response relationships have not been tested considering adherence as a continuous exposure [4, 7, 11, 13, 16]. However, a small retrospective study used receiver-operating characteristic curves to suggest that medication adherence above 88% provided the optimal sensitivity and specificity for predicting better event-free survival in a HF cohort [21]. Another retrospective HF cohort study used electronic health records to estimate a mean PDC for all HF medications, and found that for each 10% increment in mean PDC, there was a 6% and 9% decrement in hospital admissions and death respectively [5].

We have previously reported that RCS analysis can provide an useful graphical representation of the adherence-outcome relationship across the continuous adherence scale, and used to refine how PDC is categorised to predict mortality [23]. This present study extends the use of Cox regression RCS models to assess the association between RASI and β-blocker adherence as continuous exposures and long-term mortality and/or HF readmission events in a senior HF cohort. The RCS analyses confirmed an approximately curvilinear association between adherence and the risk of all-cause death and all-cause death/HF readmission for both drug classes, with a clear linear reduction in risk above a PDC of 60%. Above this point, increasing adherence was associated with significant continuous reduction in risk of all-cause death and the composite secondary outcome. Importantly, these results suggest that adherence levels to RASI or β-blockers should be targeted above the customary 80% threshold because there is no plateauing of risk reduction beyond this point. Hence, health professionals should focus on maximising medication adherence in their HF patients rather than trying to achieve an arbitrary adherence level.

Consistent with a previous study [12], we observed that patients who demonstrate high adherence to RASI and β-blocker therapy in the first year post-discharge continue to be good adherers in the subsequent year, and this may explain why they continue to have a lower long-term risk of all-cause death and/or HF hospitalisation. This finding emphasises the importance of interventions to enhance adherence as a key component of follow-up care after initial HF hospitalisation, and then ensuring that adherence is monitored and supported in the long term.

Despite significant mortality benefits, the effect of RASI or β-blocker adherence on HF hospitalisations specifically was relatively minor over the long term. This may be because precipitating factors for HF hospitalisation are diverse and medication non-adherence may only be one of many causes, including non-cardiovascular factors, for HF readmission [35]. Our unselected HF cohort is also likely to comprise a substantial number of HF patients with mildly reduced ejection fraction (HFmrEF) or preserved ejection fraction (HFpEF) among whom these pharmacotherapies may not significantly affect mortality or HF hospitalisations [2, 3]. However, there is increasing evidence that patients with HFmrEF behave more like those with HFrEF in terms of both prognosis and response to pharmacotherapies [2]. There are also observational studies and meta-analysis of randomised controlled trials that have suggested a favourable association between RASI or β-blocker therapy and subsequent mortality in patients with HFpEF, possibly through beneficial effects on comorbidities such as IHD, hypertension or diabetes [36, 37]. Furthermore, both RASI and β-blockers have prognostic benefit for secondary prevention in patients with HF of ischaemic origin.

## Strengths and limitations

We included only seniors aged 65–84 years, although this older age group and their adherence patterns are more representative of the ‘real-world’ cohort of patients with HF than those typically included in randomised clinical trials [38]. Because our study is observational, a cause-and-effect association cannot be proven. We lacked phenotype data that would have permitted covariate adjustment by clinical HF severity or ejection fraction. However, inclusion of patients with HFmrEF and HFpEF should have, if anything, biased our results towards a null effect because of an expected diminished response to RASI and β-blockers in these patients. More recently, there have been major advances in HF pharmacotherapies with introduction of the angiotensin receptor neprilysin inhibitor and sodium-glucose co-transporter-2 inhibitors [2, 3]. We suggest that similar RCS analyses should be applied to assess the adherence-outcome relationship of these new agents in future studies.

We adjusted for important sociodemographic, comorbidity and treatment factors that may have confounded the association between adherence and outcomes. Although this may not fully adjust for differences between groups, the results were very similar between covariate and propensity-adjusted analyses. However, we cannot exclude the possibility of important unmeasured cofounders and even with propensity adjustment, a healthy user bias may result in an overestimation of adherence effects [39]. Changes in treatment regimen and adherence after the landmark point might impact long-term outcomes although we found that most patients maintained their same medication adherence pattern long term [12]. Our PBS dataset contains dispensing data but not the doses prescribed and we are unable to assess if patients were on optimal dosages of medication or estimate dosage-outcome relationships. Finally, true patient compliance or consumption of the medications cannot be measured from administrative data. However, it was reasonable to assume that most patients took their dispensed medications given that the observed average number of refills for both RASI and β-blockers during the landmark period was consistent with PBS prescriptions which are intended to approximate 1-month supplies. A major strength of the study is that it is population-based with complete follow-up and capture of outcomes using person-linked administrative data.

## Conclusions

In senior patients with HF, increasing adherence to RASI and β-blockers in the first year post-HF hospitalisation was associated with reducing long-term risk of all-cause death and death/HF readmission. Importantly, RCS analysis established that an empirical adherence threshold of 80% does not provide optimal outcomes as mortality risk continues to reduce above this threshold. Since adherence to guideline-based HF pharmacotherapies is fundamental to clinical outcomes, our study reinforces the importance of interventions to optimise medication adherence as a key component of disease management programmes after HF hospitalisation. Our findings also reinforce the need for further research to reliably quantify medication adherence-outcome relationships according to diseases, medications and patient characteristics.

## Data Availability

We will consider requests for data sharing on an individual basis, with the aim to share data whenever possible for appropriate research purposes. However, this research project uses data obtained from a third-party source under strict privacy and confidentiality agreements from the Western Australian Department of Health (State) and Australian Department of Health (Federal) databases, which are governed by their ethics committees and data custodians. The data were provided after approval was granted from their standard application processes for access to the linked datasets. Therefore, any requests to share these data with other researchers will be subject to formal approval from the third-party ethics committees and data custodian(s). Researchers interested in these data should contact the Client Services Team at the Data Linkage Branch of the Western Australian Department of Health (www.datalinkage-wa.org.au/contact-us).
